# Urinary Metabolomics Study of Patients with Bicuspid Aortic Valve Disease

**DOI:** 10.3390/molecules26144220

**Published:** 2021-07-12

**Authors:** Massimo Chessa, Mario Panebianco, Sara Corbu, Milena Lussu, Angelica Dessì, Roberta Pintus, Flaminia Cesare Marincola, Vassilios Fanos

**Affiliations:** 1 Pediatric and Adult Congenital IRCCS, Policlinico San Donato, I-20097 San Donato Milanese, MI, Italy; massimo.chessa@grupposandonato.it (M.C.); mario.pb87@libero.it (M.P.); 2 Neonatal Intensive Care Unit, Azienda Ospedaliera Universitaria, University of Cagliari, S.P. n° 8, Km 0.700, I-09042 Monserrato, CA, Italy; sari.crb@gmail.com (S.C.); m.lussucagliari@gmail.com (M.L.); angelicadessi@unica.it (A.D.); gomberta@icloud.com (R.P.); vafanos@tiscali.it (V.F.); 3 Department of Chemical and Geological Sciences, University of Cagliari, I-09042 Monserrato, CA, Italy

**Keywords:** metabolomics, NMR, BAV, urine

## Abstract

Bicuspid aortic valve (BAV) is the most common congenital heart defect responsible for valvular and aortic complications in affected patients. Causes and mechanisms of this pathology are still elusive and thus the lack of early detection biomarkers leads to challenges in its diagnosis and prevention of associated cardiovascular anomalies. The aim of this study was to explore the potential use of urine Nuclear Magnetic Resonance (NMR) metabolomics to evaluate a molecular fingerprint of BAV. Both multivariate and univariate statistical analyses were performed to compare the urinary metabolome of 20 patients with BAV with that of 24 matched controls. Orthogonal partial least squared discriminant analysis (OPLS-DA) showed statistically significant discrimination between cases and controls, suggesting seven metabolites (3-hydroxybutyrate, alanine, betaine, creatine, glycine, hippurate, and taurine) as potential biomarkers. Among these, glycine, hippurate and taurine individually displayed medium sensitivity and specificity by receiver operating characteristic (ROC) analysis. Pathway analysis indicated two metabolic pathways likely perturbed in BAV subjects. Possible contributions of gut microbiota activity and energy imbalance are also discussed. These results constitute encouraging preliminary findings in favor of the use of urine-based metabolomics for early diagnosis of BAV.

## 1. Introduction

Bicuspid aortic valve (BAV) is the most common congenital heart defect, usually asymptomatic, resulting from an abnormal aortic cusp formation during valvulogenesis. It has a prevalence of 0.5 to 2% in the general population [1,2] and 30%, approximately, in individuals with aortic valve disorders [3,4]. Generally, males are more often affected than females.

BAV is considered a multifaceted heterogeneous disease because of the diverse clinical manifestations [5,6,7]. During the last decades, the knowledge of genetic and epigenetic aspects of BAV has increased significantly [8]; however, elucidating the mechanisms underlying BAV and related complications still poses several challenges. In clinical practice, its diagnosis is mainly entrusted to imaging techniques, and no effective strategies exist to prevent progression and its related disorders. In this regard, due to the association of BAV with lifelong increased risk of adverse cardiovascular events, among which the most insidious is bicuspid aortopathy [9], there is a growing need for developing new screening methods and identification of non-invasive biomarkers for improving the prompt diagnosis and monitoring of BAV, and to define the best and safest time point for surgery.

Metabolomics is the study of the biochemical profile of the metabolome, i.e., the set of low molecular weight (<1.5 kDa) metabolites in a biological system arising from physiological and pathological cellular processes [10]. The characterization of the metabolome makes metabolomics a promising strategy to identify biomarkers in clinical practice for the diagnosis and assessment of severity and response to therapy in a number of clinical disease states [11]. This approach has provided valuable insight also into the metabolic changes associated with cardiovascular diseases [12] such as myocardial ischemia [13] and infarction, coronary artery disease [14], and heart failure [15]. To the best of our knowledge, only a few metabolomics studies have been performed to identify metabolic alterations in BAV patients [16,17,18] with a special focus on the lipid molecular class, it being the dysregulated lipid metabolism recognized as an established risk factor in cardiovascular diseases [19].

In the current study, we explored the potential of urine metabolome analysis as an informative tool to support BAV diagnosis. Urine is a biofluid largely used for clinical diagnosis since it contains not only many plasma components but also the catabolic products of different metabolic pathways. Compared to other biofluids, it offers different advantages: it is abundant, readily available, easy to store, and non-invasively collected. By a comparative nontargeted metabolomics analysis of urine from BAV patients and matched controls based on proton Nuclear Magnetic Resonance (^1^H NMR) spectroscopy, we searched for a possible characteristic metabolite signature associated with BAV.

## 2. Results

Our study population comprised 44 individuals (20 cases and 24 controls) including 31 men and 13 women between the ages of 18 and 74 years. In order to reduce uninformative variations that could interfere with the identification of relevant information encoded in the experimental spectral data set, controls were selected according to some matching variables, and restricted exclusion criteria for the health status of subjects were applied to both groups (see Materials and Methods section). The baseline characteristics of the study population are summarized in Table 1. Only the body surface area (BSA) was significantly higher in BAV than controls.

All BAV subjects were asymptomatic. Echocardiographic data are shown in Table 2. The morphologic analysis of BAV showed that 12 patients (60%) had type 1 and eight patients (40%) type 2 BAV. Compared to controls, the patient group showed no significant dilatation of the aortic dimensions above the normal values [20]. Similarly, LV indices of the BAV group were significantly higher than in controls but still within the normal range [20].

**Figure 1:**
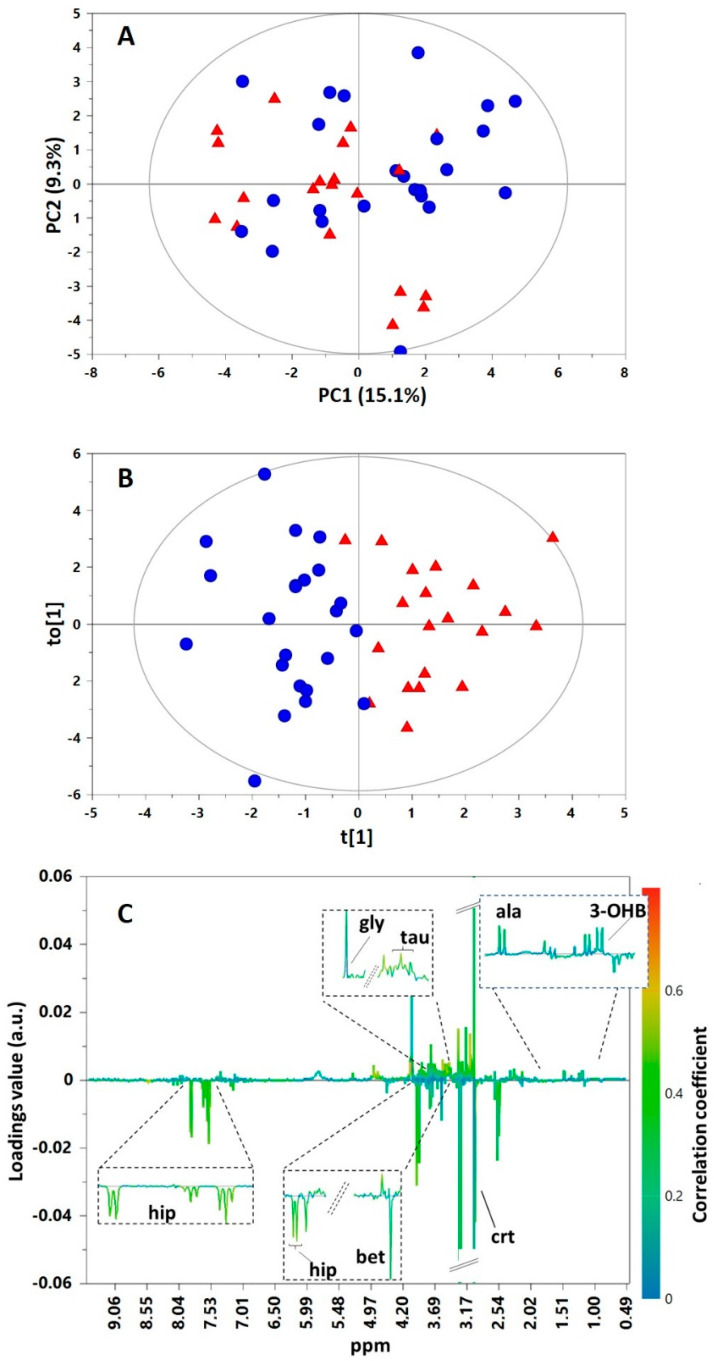
PCA scores plot (**A**), OPLS−DA scores plot (**B**), and correlation coefficient S-line plot (**C**) for the pairwise comparison between BAV (▲) and controls (●). Abbreviation: 3-OHB, 3-hydroxybutyrate; ala, alanine; bet, betaine; crt, creatine; gly, glycine; hip, hippurate; tau, taurine.

An untargeted metabolomics approach was employed to compare the NMR urinary metabolic profiles of BAV and controls. The unsupervised analysis of the entire data set in the form of PCA provided a model with two components explaining around 24% of the spectral variance. Visual inspection of the scores plot resulted in no detection of either outliers (i.e., scores outside the Hotelling’s T2 range) or clustering based on the disease status (Figure 1A). To further explore whether any urinary compositional variability related to this congenital malformation was present in BAV subjects compared to controls, a supervised analysis was carried out by using OPLS-DA. This approach led to a good separation between groups (Figure 1B) with acceptable values of explained variation related to classes (*R*^2^Y = 0.78) and predictive ability (*Q*^2^ = 0.46), thus indicating reliable changes in urine metabolic profiles of BAV in comparison with controls. Additionally, a CV-ANOVA test revealed that the model was significant (*p* = 0.003), and a permutation test for the Y variable indicated that it was not influenced by overfitting (Y-axis intercept equal to 0.318 for *Q*^2^). Furthermore, receiver operating characteristic (ROC) analysis of the model revealed an AUC equal to 0.863. To further validate these results, an external validation test was done by randomly taking out 25% of samples in the data set as blind samples and processing the OPLS-DA prediction model with the remaining 75% of samples. This operation, repeated 20 times, provided an average correct classification rate of 0.70 ± 0.05 (Fischer *p*-value < 0.05).

The analysis of the OPLS-DA correlation coefficient S-line plot suggested that the BAV metabolic signature primarily responsible for class separation comprised a panel of seven metabolites (Figure 1C). In particular, the levels of urinary 3-hydroxybutyrate (3-OHB), alanine, glycine, and taurine were higher in cases than in controls, while betaine, creatine, and hippurate were less abundant.

**Figure 2:**
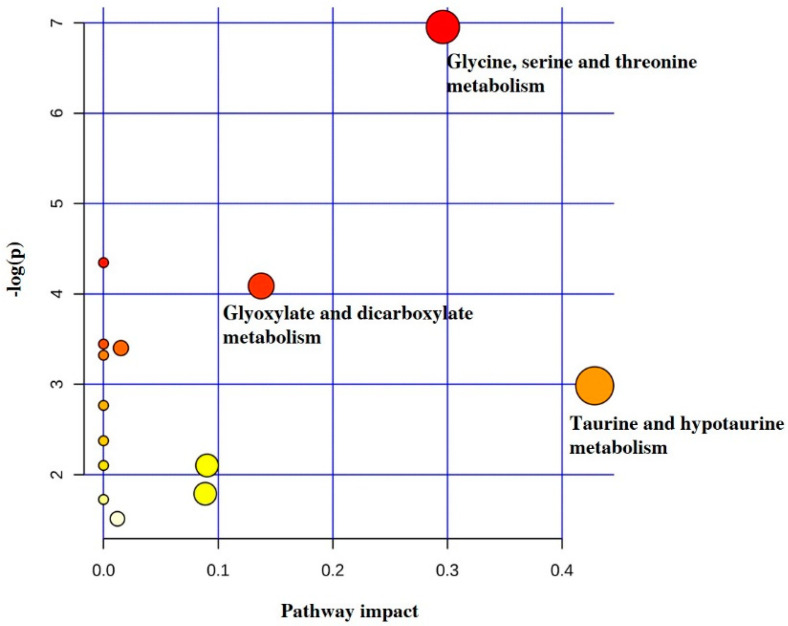
Topology map, generated using MetaboAnalyst, describing the impact of the seven discriminant metabolites identified through comparative analysis of BAV against controls by OPLS-DA. Bubble size is proportional to the impact of each pathway, while bubble color denotes the degrees of significance from highest (red) to lowest (white).

The specific set of discriminant metabolites found in a pairwise comparison was submitted to pathway analysis by using the MetPA module in order to map the possible metabolic implications of the detected differences. As shown in Figure 2, the seven biomarkers were involved in 13 metabolic pathways whose weight was judged by their influence value. Only two metabolic pathways (glycine, serine and threonine metabolism, and taurine and hypotaurine metabolism) had an impact value > 0.1, and thus they were screened as potential target pathways mostly related to BAV.

**Figure 3:**
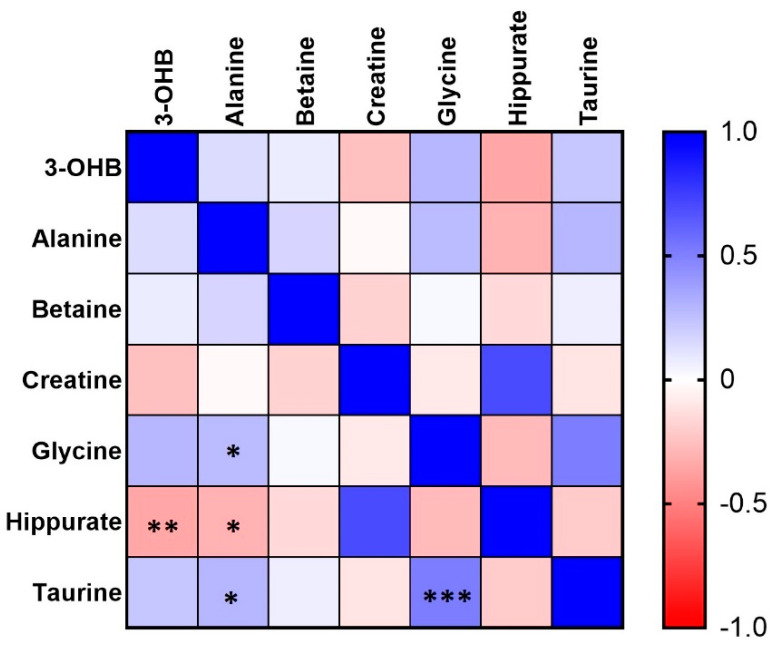
Heatmap representing the correlation coefficient matrix (reflecting Pearson’s correlation coefficients) between urine metabolites identified as potential biomarkers differentiating BAV from controls. * *p* < 0.05; ** *p* < 0.01; *** *p* < 0.001.

We also assessed the relationship between the abovementioned putative biomarkers (Figure 3). The largest positive correlation was observed between taurine and glycine (*r* = 0.553, *p* = 3.7·10^−4^). The correlation between hippurate and 3-OHB was also significant but negative (*r* = −0.350, *p* = 0.02). Furthermore, a weak but significant (*p* < 0.05) correlation was observed for alanine with glycine, hippurate, and taurine.

**Figure 4:**
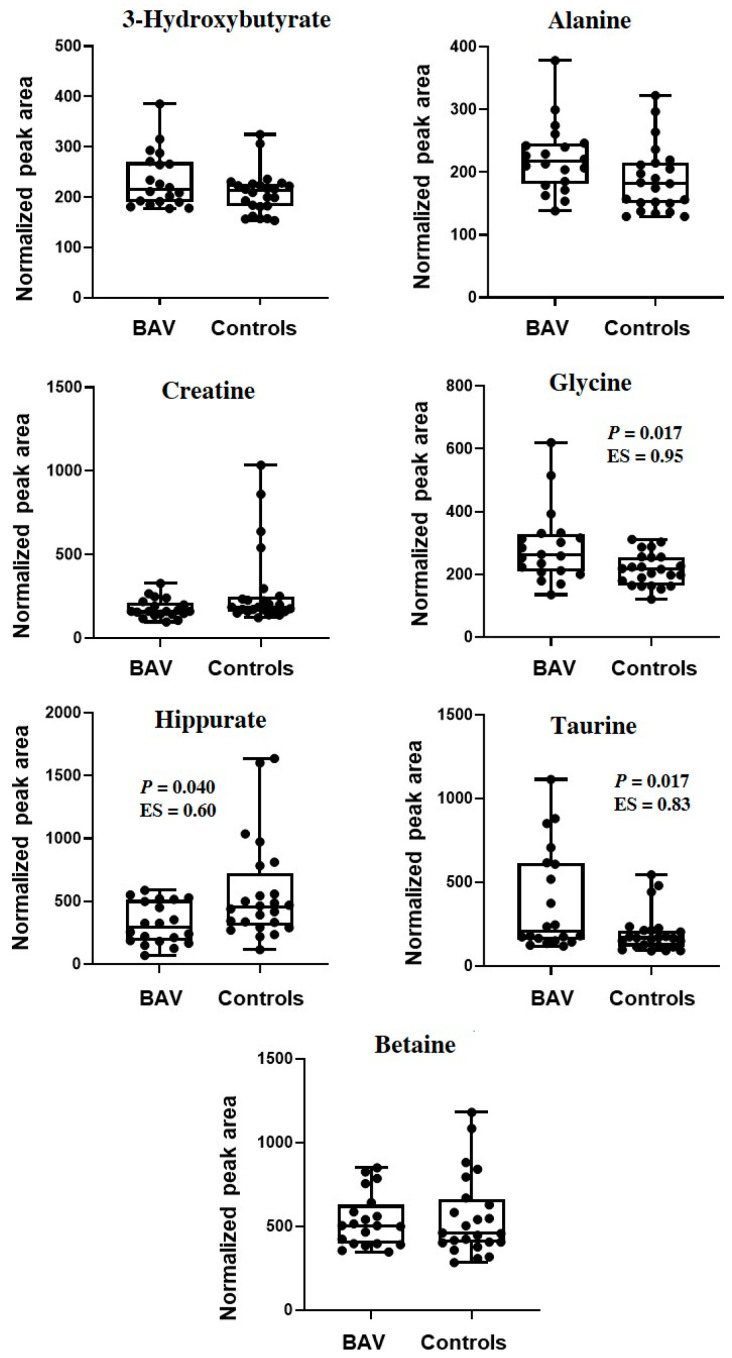
Box-and-whisker plots of the relative content of metabolites that were significantly perturbed in BAV compared to controls as indicated by the OPLS-DA analysis of the NMR data set. Quantification was achieved after the calculation of the area under the corresponding peaks. The boxes show the median and the inter-quartile range for each metabolite in the two groups. Abbreviation: ES, effect size value.

**Figure 5:**
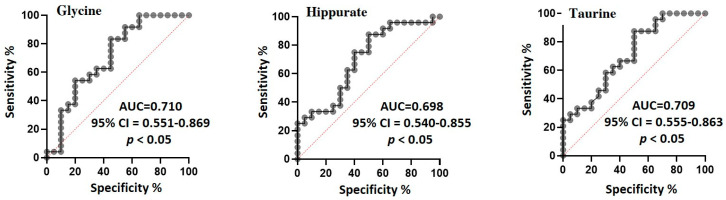
Receiver operating characteristic (ROC) analysis of glycine, hippurate, and taurine for differentiating BAV patients from healthy controls.

It is worth stating that the multivariate statistical analysis methods for analysis of metabolomics data focus on the relationships among variables (i.e., metabolites). Thus, the contents of the seven markers discriminating between cases and controls were also screened individually using univariate statistical analysis. To this aim, by the integration of the corresponding selected NMR signals, the variation in the relative content was monitored and depicted in the box plots in Figure 4. Of the seven compounds, three exhibited a significant inter-group variation (*p* < 0.05), namely, glycine, taurine, and hippurate, with large absolute effect sizes for the first two molecules (>0.8) and a medium value for the latter (>0.5). These three candidate biomarkers were also subjected to ROC analysis to evaluate their ability to separate BAV and controls (Figure 5). The statistical parameters of the ROC curves showed an AUC value ≥0.70 for all of three metabolites, thus pointing out a medium diagnostic capability for each compound. It is worth noting that the AUC generated with each metabolite was lower than those of the curve generated from the entire data set by OPLS-DA, indicating an improvement of the diagnostic performance of the multivariate statistical model.

## 3. Discussion

In the last decades, metabolomics has emerged as a useful tool in the study of diseases and a fascinating and innovative method toward the understanding of pathogenesis, diagnosis and management of different pathologies, and the development of tailored approaches [21,22]. Blood and urine are both suitable biofluids for metabolomics analysis. On one hand, blood (plasma or serum) offers the main advantage of being uniquely uniform and very homeostatic. Thus, it is less affected by confounding factors such as age, gender, or diet. On the other, urine is easier to obtain and handle, samples need less preparation, and the collection is not invasive. In cardiovascular medicine, metabolomics studies have been focused principally on the analysis of human plasma or serum [23,24,25]. In the particular case of the bicuspid aortic valve (BAV) defect, the metabolic snapshots generated by metabolomics studies on plasma from BAV subjects have highlighted perturbations in purine metabolism and fatty acid biosynthesis, redox imbalance, and deficient energy production [16,18].

Various studies have shown that urine NMR metabolomics has potential as a screening tool for accurate diagnosis [26,27,28]. Since, to the best of our knowledge, no investigation has been performed so far on the urine metabolome of BAV subjects, in the present study we applied urine-based metabolomics for a comparative analysis of the metabolic profile of adult asymptomatic subjects with BAV (*n* = 20) and healthy controls (*n* = 24). In order to reduce age and gender dependence heterogeneity in the study population, age and gender matching between cases and controls was applied. The multivariate statistical analyses of the NMR spectral data suggested seven discriminant metabolites, three of which (hippurate, taurine, and glycine) individually exhibited medium diagnostic power by ROC analysis. In particular, we found low levels of hippurate (Hip) in BAV. Hip is the glycine conjugate of benzoic acid resulting from the metabolic conversion by gut microbes of dietary aromatic compounds. Therefore, perturbations in Hip urinary levels are often attributed to changes in gut microbial activities [29]. Accordingly, our result could be taken as an indication of a possible association between gut microbial and BAV disease. Consistent with this view, there is the accumulating evidence of a contribution of gut microbiota to the development of cardiovascular diseases [30]. A further indication supporting this hypothesis may arise from the lower content of betaine in BAV compared to controls. Indeed, besides being involved in several important metabolic pathways, including the formation of specific phosphatidylcholines (lipid metabolism) and the synthesis of homocysteine (a metabolite with a debated association with cardiovascular morbidity and mortality [31]), betaine is a metabolite associated with gut microbial activity [32]. It can produce trimethylamine under the effect of gut microbiota, which is further oxidized as trimethylamine-N-oxide (TMAO) in liver, another metabolite having dose-dependent associations with cardiovascular diseases [33,34].

Additionally, our data evidenced a higher excretion of taurine (Tau) and 3-hydroxybutyrate (3-OHB) in BAV patients compared to controls. Tau is a nonessential amino acid, derived primarily from the diet and produced in very limited quantities by the heart and brain [35]. It has multiple vital functions in cardiac processes, contributing to the regulation of blood pressure, adequate oxidative metabolism, and ATP production [36]. Furthermore, most of the beneficial effects of Tau on cardiovascular diseases have been suggested to be due to its action on reactive oxygen species as well as on intracellular Na^+^ and Ca^2+^ overloads [37]. 3-OHB is a ketone body, synthesized from oxidation of fatty acids (FAs) primarily in the liver. Ketone bodies are energy substrates for heart-like fatty acids, glucose, lactate, and amino acids. It has been shown that alterations in ketone bodies can be associated with heart disease as a consequence of an imbalance of lipid oxidation, although not always do these anomalies follow the same trend. For instance, higher levels of ketone bodies in blood from heart failure (HF) patients were observed compared to healthy controls [38,39]. On the contrary, a recent metabolomics investigation showed that the serum concentrations of the ketone bodies were lower in patients with HF with reduced ejection fraction than in controls [40]. Although the physiological importance of these metabolomic findings has not been determined yet, we do not exclude that the higher urinary level of 3-OHB in BAV compared to controls may also be indicative of a possible derangement of energy metabolism, as also suggested by plasma-based metabolomics studies on BAV [16,17]. The disturbance of energy supply would explain also the lower levels of creatine (Cr) in BAV than controls. Indeed, Cr is a metabolite essential for normal cardiac function. It might augment cellular energy supply and attenuate the loss of cellular Ca^2+^ whose abnormal modulation is known to be implicated in various cardiac pathologies [41].

It has been reported that abnormalities in amino acid metabolism are associated with cardiovascular diseases [42]. The results of our study showed that the levels of two amino acids, alanine (Ala) and glycine (Gly) were more elevated in BAV urine than in controls. Ala is a glycogenic amino acid that can be converted into glucose by gluconeogenesis, acting as an energy source to meet the huge demand of energy consumed in various metabolic activities. Gly is a non-essential amino acid, participating in a wide range of metabolic pathways [43]. It can regulate lipid metabolism [44] and immune cell activation [45]. Furthermore, it is a substrate for the biosynthesis of glutathione, a major antioxidant in human cells [46]. Recently, its circulating levels have also been associated with a lower incidence of myocardial infarction [44]. The results of our metabolic pathway analysis, in particular, suggested a possible perturbation of glycine metabolism in BAV, a pathway producing important energy metabolism precursors for the Krebs cycle. Nevertheless, since Gly is a metabolite at the intersection of many metabolic pathways, we cannot rule out that the higher levels of Gly in BAV compared to controls may represent also the causal effect of a metabolite to which glycine is metabolically closely linked.

## 4. Strengths and Limitations of the Study

This study has weaknesses and strengths. The main criticism is represented by the small sample size, thus limiting the statistical power of the results. At least 30 subjects per group should be included in clinical metabolomics studies to successfully achieve discriminatory identification of metabolite profiles, although this number is dependent on design details. However, it should be remembered that the present study was intended as explorative, aimed at investigating the potential of urine metabolome analysis as an informative tool to support BAV diagnosis. Based on the low number of samples, it can thus pilot further studies with larger sample sizes to determine the clinical utility of urine NMR in the diagnosis of BAV.

While somewhat limited, the present study has also an important strength. To the best of our knowledge, there are no previously published data on the urine metabolome of BAV subjects. A limited number of metabolomics studies have been performed on BAV. Among these, Wang et al. analyzed the serum metabolome of BAV patients and healthy individuals by liquid chromatography–mass spectrometry (LC-MS) [16]. Doppler et al. compared the ascending thoracic aortic wall tissue of BAV patients with aortic aneurysms, tricuspid aortic valve (TAV) patients with aortic aneurysms, and TAV subjects undergoing surgery for aortic dissection [17]. The analytical technique was the Flow Injection Analysis Tandem Mass Spectrometry (FIA-MS/MS). Martinez et al. analyzed the plasma metabolic profile of TAV individuals, BAV patients with no aortic dilation, BAV patients with ascending aorta dilation at the time of diagnosis, and patients with TAV and ascending aorta dilation [17]. Another study was performed by Xiong et al. with a specific focus on the metabolomics profile involved in BAV aortic stenosis prior to and after transcatheter aortic valve replacement in comparison with TAV [47]. Although the abovementioned investigations vary to great extend in terms of study design, their results point to the hypothesis of an important role of the lipid and energy metabolisms in BAV disease. Complementing this information, our findings support the presence of altered energy metabolism under the BAV condition and suggest a potential role of intestinal microbiota in this cardiac abnormality. In light of the increasing evidence of a possible relationship of gut microbiota and cardiovascular disease development [30], this association deserves to be investigated further, even in the case of BAV malformation.

## 5. Materials and Methods

### 5.1. Study Population and Sample Collection

This prospective, monocentric study included 44 adult subjects (20 cases and 24 healthy controls) enrolled at the Paediatric and Adult Congenital Heart Centre, IRCCS Policlinico San Donato, Milan, Italy. The institution’s Ethics Board approved the study (CE 126/INT/15) and informed consent was obtained from the participants.

Each subject underwent a complete physical examination (height, weight, heart rate, blood pressure [BP]), a cardiac examination performed by professional cardiologists, and a 2-dimensional (2D) transthoracic color Doppler echocardiography (TTE) to address the diagnosis of BAV. BP was measured before or after the ultrasound examination using a validated automated oscillometric. Exclusion criteria were: diagnosed or suspected connective tissue disorders such as Marfan syndrome, previous cytoreductive treatments (i.e., chemotherapy, radiotherapy), lasting steroidal therapy, graft-versus-host disease, active infective diseases, serological positivity for HIV, hepatitis B virus (HBV) (in non-vaccinated subject) and/or hepatitis C virus (HCV), and presence of other major congenital heart defects (e.g., aortic coarctation, aortic valve surgery, aortic stenosis, aortic insufficiency, or thoracic aortic aneurysm).

Personal medical history (i.e., comorbidities, previous cardiac surgical or hemodynamic interventions, age at BAV diagnosis, symptoms), the use of medications, physical activity, and family history of congenital heart diseases, especially aortic valve diseases, were investigated.

Urine samples were self-collected by participants under overnight fasting conditions following provided instructions and immediately delivered to the laboratory on ice. Once in the laboratory, samples were stored at −80 °C until shipping on dry ice to the University of Cagliari for metabolomics analysis.

### 5.2. Chemicals

Sodium 3-trimethylsilyl [2,2,3,3-*d*4]-propionate (TSP) and deuterium oxide (D_2_O, 99.9% D) were purchased from Cambridge Isotope Laboratories, Inc. (Cambridge, MA, USA). Sodium azide (NaN_3_), K_2_HPO_4_·3H_2_O and KH_2_PO_4_, all at analytical grade, were obtained from Sigma Aldrich (Milano, Italy).

### 5.3. Sample Preparation

Urine samples were thawed in ice. An aliquot of 1 mL was transferred into a microcentrifuge tube and 10 μL of NaN_3_ (10% *w*/*w*) was added to avoid possible bacterial growth. The samples were vortexed and then centrifuged at 12,000 *g* for 10 min at 4 °C. To stabilize the pH of urine samples, 630 μL of the supernatants were mixed with 70 μL of phosphate buffer solution in D_2_O (1.5 M, pH 7.4) containing TSP (6 mM) as internal standard. The mixture was vortexed and an aliquot of 650 μL was transferred into a 5 mm NMR tube for the analysis.

### 5.4. ^1^H NMR Spectroscopy

^1^H NMR spectra were recorded at 300 K using a Varian Unity Inova 500 NMR spectrometer (Agilent Technologies, Santa Clara, CA, USA), operating at 499.839 MHz. Water suppression was achieved with a Noesypresat pulse sequence during a 3.5 s relaxation delay and 1 ms mixing time. NMR spectra were acquired with an acquisition time of 1.5 s, 32 K data points and 256 scans over a spectral width of 6000 Hz.

NMR spectra were processed by using MestReNova (Version 10.0, Mestrelab Research SL, Valencia, Spain). After Fourier transformation with 0.3 Hz line broadening and a zero-filling to 64 K, spectra were phased and baseline corrected, and the chemical shift scale was set by assigning a value of δ = 0.00 ppm to the signal of the internal standard TSP. After correction for misalignments in chemical shift primarily due to pH-dependent signals, NMR spectra were binned to 0.002 ppm intervals between 0.5 and 9.5 ppm, excluding the region corresponding to water (4.6–5.2 ppm) and TSP (−0.5 ppm) signals. All spectra were normalized using the Probabilistic Quotient Normalization (PQN) method [48].

### 5.5. Statistical Analysis

The binned NMR data set was converted into a Microsoft Office Excel file and then imported into SIMCA version 16.0 (Umetrics, Umeå, Sweden) for statistical assessment. Prior to multivariate statistical analysis, data were Pareto scaled to emphasize both large- and small-concentration metabolite signals. Then, principal component analysis (PCA) [49] and orthogonal partial least square-discriminant analysis (OPLS-DA) [50] were applied. PCA, an unsupervised pattern recognition method, was first performed to examine the intrinsic variation in the data set. Afterwards, OPLS-DA was performed to maximize the separation between groups of observations by removing variability not relevant to class separation. The model performance was evaluated by the coefficients *R*^2^ and *Q*^2^, indicating goodness of fit and prediction, respectively. To provide a further objective assessment of the performance and stability of the model, internal validation was performed by cross validation-analysis of variance (CV-ANOVA), with a *p*-value < 0.05 [51] and permutation testing to check the validity and the degree of overfitting. The predictive accuracy of the OPLS-DA models was also examined by receiver operating characteristic (ROC) analysis [52]. The ROC is a calculation of the rate of false positive (1-sensitivity) to true positive (specificity) classifications that makes no assumption of class boundaries. The area under the ROC curve (AUC) is equal to the probability that reflects the overall diagnostic accuracy of a certain index in the diagnosis of diseases. An AUC lower than 0.5 indicates a classification accuracy no better than chance, 0.5  <  AUC  ≤  0.7 means low diagnostic accuracy, 0.7  <  AUC  ≤  0.9 means medium diagnostic accuracy, and 0.9  <  AUC < 1.0 means high diagnostic accuracy.

For validation purposes, external validation of the OPLS-DA models was also applied by dividing the data set in such a way that randomly selected samples of 75% of the participants were used to form a training set, while the samples of the remaining 25% were used as the test set. This procedure was repeated 20 times to construct 20 random training-test set pairs. OPLS-DA models were built based on the training set and then blindly predicted the classes of the samples in the test set. Each time the correct classification rate was calculated.

The contribution of variables to group discrimination was evaluated by analyzing the OPLS-DA correlation coefficient loading plots (S-line). This plot depicts simultaneously the outcomes of the covariance (peak height) and correlation (color code) analyses applied to the ^1^H-NMR dataset. The observed phase of the resonance signals represents the relative changes in the concentration of metabolites: positive peak phase reflects metabolites with increased concentration in the group positioned along the positive direction of the first component of the model, and vice versa. The color of variables contains information on their statistical significance: a hot color shows a more significant contribution than the cold one for the intergroup discrimination, as calculated from the correlation matrix. The spectral regions that contributed most to class separation were selected according to the following criteria: |p(cov)| ≥ 0.05; |p(corr)| ≥ 0.5. Metabolites identification was performed according to the literature [53,54], the Human Metabolome Database (www.hmdb.ca, accessed on 28 May 2021), and by using the trial version of Chenomx NMR suite 7.0 (Chenomx Inc., Alberta, AB, Canada).

Pathway analysis was performed using Metabolomics Pathway Analysis (MetPA; https://www.metaboanalyst.ca, accessed on 28 May 2021), a web-based metabolomics tool [55] that combines several advanced pathway enrichment analysis procedures along with the analysis of pathway topological characteristics to help the identification of the most relevant metabolic pathways involved in a given metabolomic study.

Metabolites identified by discriminant loadings in the OPLS-DA S-line plot were quantified by analyzing the integrals of selected distinctive NMR signals. Relative contents in the two groups were submitted to univariate statistical analysis performed using GraphPad Prism version 8.0.0 (GraphPad Software, San Diego, California, CA, USA, www.graphpad.com, accessed on 28 May 2021). The Mann–Whitney U test was used to assess group differences between metabolites (a *p*-value < 0.05 was evaluated statistically significant) with effect sizes (ES) calculated by Cohen’s d-test [56]. ES values between 0.2 and 0.5 were classified as small, between 0.5 and 0.8 as medium, and over 0.8 as large. ROC curves were built to test the sensibility and the specificity of the selected metabolites by using the same software. Correlation analysis was assessed using a Pearson’s test.

## 6. Conclusions

Many individuals with BAV do not experience symptoms until they begin to have complications. Imaging techniques are the current standard for BAV diagnosis. However, this is done when symptoms have already manifested. Thus, early diagnosis is fundamental to preventing BAV symptoms and the adverse cardiac outcomes related to this heart defect. The discovery of candidate biomarkers of BAV can contribute to developing preventive interventions and to improving the understanding of the structural molecular basis of this pathology.

Our study allowed a preliminary evaluation of the potential utility of urine NMR metabolomics to provide new insights into BAV. Overall, the findings suggest that BAV has a measurable influence on the urinary metabolome of patients. A panel set of seven potential discriminant metabolites was identified by multivariate statistical analysis, suggesting a possible association of BAV with two metabolic pathways (taurine and hypotaurine metabolism, and glycine, serine and threonine metabolism), gut microbial activity, and energy imbalance. Furthermore, three metabolites (glycine, taurine, and hippurate) individually showed a medium diagnostic potential. These findings can contribute to a better understanding of the pathology of BAV and provide a basis for future investigations on the use of noninvasive urinary metabolomics for improved diagnosis and prognosis of this complex and clinically heterogeneous disease.

## Figures and Tables

**Table 1:** Baseline characteristics of the study population: cases vs. controls.

	BAV	Controls	*p*-Value
Number	20	24	-
Age, yrs	40.1 (18–74)	42.7 (23–69)	0.600
Males, *n* (%)	17 (85)	14 (58)	0.055
Weight, kg	75.15 ± 7.47	71.8 ± 8.1	0.169
Height, cm	175 (152–189)	177 (158–186)	0.389
BMI, kg/m^2^	24.60 ± 2.48	23.36. ± 1.88	0.687
BSA_DB,_ m^2^	1.90 ± 0.12	1.79 ± 0.14	0.014
HR, bpm	69.95 ± 11.76	72.28 ± 11.85	0.514
Smoking, *n* (%)	5 (25)	6 (25)	1.00

Continuous normally distributed variables are reported as mean ± standard deviation; continuous non-normally distributed variables are reported as median (interquartile range, IQR); categorical variables as *n* (%). Abbreviations: BMI, body mass index; BSA_DB_, body surface area, calculated according to DuBois’s formula; HR, heart rate.

**Table 2:** Echocardiographic measurements of aortic root dimensions and left ventricular volume.

	BAV (*n* = 20)	Controls (*n* = 24)	*p*-Value
*Aortic root*			
Annulus (mm)	23.6 (20–26)	21.83 (19–25)	0.023
Sinuses of Valsalva (mm)	28.5 (28–34)	26.12 (25–28)	0.015
Sinotubular junction (mm)	27.54 ± 3.87	25.29 ± 2.31	0.024
Ascending Aorta (mm)	28.65 ± 4.3	26.20 ± 2.63	0.021
*Left ventricular*			
ESV index (mL/m^2^)	21.85 ± 6.19	17.90 ± 3.88	0.014
EDV index (mL/m^2^)	61.95 ± 9.98	54.29 ± 10.12	0.015
SV index (mL/m^2^)	39.49 ± 7.67	32.83 ± 9.63	0.043
EF (%)	61.71 ± 8.44	64.04 ± 4.73	0.406
*BAV morphology*			
Type 1, *n* (%)	12 (60)	-	-
Type 2, *n* (%)	8 (40)	-	-

Continuous normally distributed variables are reported as mean ± standard deviation; continuous non-normally distributed variables are reported as median [interquartile range (IQR)]; categorical variables as *n* (%). Abbreviations: EDV, end-diastolic volume; EF, ejection fraction; SV, stroke volume; ESV, end-systolic volume; Type 1, fusion of the left coronary cusp and right coronary cusp (L-R); Type 2, fusion of the right coronary cusp with the non-coronary cusp.

## Data Availability

The data presented in this study are available on request from the corresponding author.

## Sample Availability

Samples of the compounds are not available from the authors.
